# Systematically characterizing dysfunctional long intergenic non-coding RNAs in multiple brain regions of major psychosis

**DOI:** 10.18632/oncotarget.12122

**Published:** 2016-09-19

**Authors:** Jing Hu, Jinyuan Xu, Lin Pang, Hongying Zhao, Feng Li, Yulan Deng, Ling Liu, Yujia Lan, Xinxin Zhang, Tingting Zhao, Chaohan Xu, Chun Xu, Yun Xiao, Xia Li

**Affiliations:** ^1^ College of Bioinformatics Science and Technology, Harbin Medical University, Harbin, Heilongjiang, China; ^2^ Department of Neurology, The First Affiliated Hospital of Harbin Medical University, Harbin, Heilongjiang, China; ^3^ Department of Psychiatry, Texas Tech University Health Science Center, El Paso, Texas, United States of America

**Keywords:** long intergenic non-coding RNA, schizophrenia, bipolar disorder, brain region, RNA sequencing, Pathology Section

## Abstract

Schizophrenia (SZ) and bipolar disorder (BD) are severe neuropsychiatric disorders with serious impact on patients, together termed “major psychosis”. Recently, long intergenic non-coding RNAs (lincRNAs) were reported to play important roles in mental diseases. However, little was known about their molecular mechanism in pathogenesis of SZ and BD. Here, we performed RNA sequencing on 82 post-mortem brain tissues from three brain regions (orbitofrontal cortex (BA11), anterior cingulate cortex (BA24) and dorsolateral prefrontal cortex (BA9)) of patients with SZ and BD and control subjects, generating over one billion reads. We characterized lincRNA transcriptome in the three brain regions and identified 20 differentially expressed lincRNAs (DELincRNAs) in BA11 for BD, 34 and 1 in BA24 and BA9 for SZ, respectively. Our results showed that these DELincRNAs exhibited brain region-specific patterns. Applying weighted gene co-expression network analysis, we revealed that DELincRNAs together with other genes can function as modules to perform different functions in different brain regions, such as immune system development in BA24 and oligodendrocyte differentiation in BA9. Additionally, we found that DNA methylation alteration could partly explain the dysregulation of lincRNAs, some of which could function as enhancers in the pathogenesis of major psychosis. Together, we performed systematical characterization of dysfunctional lincRNAs in multiple brain regions of major psychosis, which provided a valuable resource to understand their roles in SZ and BD pathology and helped to discover novel biomarkers.

## INTRODUCTION

Schizophrenia (SZ) and bipolar disorder (BD), together termed “major psychosis”, both affect approximately 1% of the world's population and lead to severe impact on the life quality of patients [1]. Previous studies of SZ and BD mostly focused on the genetic factors and identified several disease-associated risk genes or variations [2, 3]. High-throughput analysis of gene expression profiles also provided additional insight into the potential biological processes implicated in SZ and BD [4]. However, the biological mechanisms underlying the pathophysiology of the disorders still need further investigation.

RNA-seq-based transcriptome analyses uncovered a large number of long intergenic non-coding RNAs (lincRNAs) that are a class of important regulatory molecules in gene expression and various diseases [5, 6]. LincRNAs display prominently spatial and temporal expression and show highly specific expression in the brain [7]. Accumulating evidence suggests that lincRNAs are implicated in brain evolution, neural development and cognitive processes [8]. Recently, the important roles of lincRNAs are also revealed in SZ and BD. For example, *Gomafu*, a lncRNA that is associated with alternative splicing, was found to contribute to pathogenic splicing of SZ-associated genes *DISC1* and *ERBB4* in SZ patient brains [9]. Akula et al. analyzed expression profiles of BD patients and identified a differentially expressed lincRNA *LINC00173* in BD [10]. However, the understanding of lincRNAs in major psychosis is still in its infancy. A systematical characterization of lincRNA transcriptome may help us better understand the pathological mechanism of SZ and BD.

In this study, we performed RNA sequencing on three brain regions, Brodmann's Area (BA) 11, BA24 and BA9, of 82 postmortem brain samples from SZ and BD patients to explore the landscape of lincRNAs in SZ and BD. We characterized the lincRNA transcriptomes of these three brain regions and identified numerous differentially expressed lincRNAs (DELincRNAs). These DELincRNAs exhibited strong brain region-specific expression change patterns. We identified three dysfunctional lincRNA modules based on weighted gene co-expression network analysis (WGCNA), revealing that DELincRNAs cooperating with important protein-coding genes participate in distinct biological processes in different brain regions. By analyzing DNA methylation levels and histone modification, the dysfunctional mechanisms underlying differential expression of lincRNAs were further characterized.

## RESULTS

### Summary of patient demographics

We performed RNA sequencing on three brain regions namely the BA11 (part of orbitofrontal cortex), BA24 (part of anterior cingulate) and BA9 (part of dorsolateral prefrontal cortex) from SZ and BD patients and psychiatrically normal individuals. The demographics data of samples used in this study were listed in Table 1. In summary, there were 44 BA11 samples from 16 SZ, 16 BD and 12 control subjects, and 19 BA24 and 19 BA9 samples from the same subjects including 6 SZ, 7 BD and 6 controls. There were no significant differences in sex, age, postmortem interval (PMI), race or brain PH between cases (SZ or BD) and controls (*P*-value>0.05, t test for continuous traits and Fisher's exact test for categorical traits).

**Table 1:** Summary of demographics for samples used in this study

Brain Region	Demographics	Control	SZ	BD	*P*-value
BA11	Number of Tissue Sample	12	16	16	
	Sex (males/females)	9/3	11/5	8/8	1.0 (SZ) ; 0.25 (BD)
	Age at sample collection	41.8 ± 6.3	39.3 ± 8.9	47.7 ± 9.7	0.40 (SZ) ; 0.06 (BD)
	Age at onset	NA	23.9 ± 6.2	25.1 ± 7.4	
	Postmortem Interval (PMI)	30.2 ± 14.2	37.9 ± 18.0	41.9 ± 21.3	0.21 (SZ) ; 0.09 (BD)
	Race (w/n)	12/0	16/0	15/1	
	BrainPH	6.65 ± 0.31	6.49 ± 0.22	6.48 ± 0.20	0.14 (SZ) ; 0.12 (BD)
BA24	Number of Tissue Sample	6	6	7	
	Sex (males/females)	5/1	4/2	4/3	1.0 (SZ) ; 0.56 (BD)
	Age at sample collection	46.5 ± 16.0	55 ± 4.6	46.6 ± 6.0	0.26 (SZ) ; 0.99(BD)
	Age at onset	NA	25.7 ± 8.1	24 ± 7.4, NA(2)a	
	Postmortem Interval (PMI)	24.3 ± 5.1	30.3 ± 5.3	27 ± 7.0	0.07 (SZ) ; 0.44 (BD)
	Race (w/h)	5/1	4/2	5/2	1.0 (SZ) ; 1.0 (BD)
BA9	Number of Tissue Sample	6	6	7	
	Sex (males/females)	5/1	4/2	4/3	1.0 (SZ) ; 0.56 (BD)
	Age at sample collection	46.5 ± 16.0	55 ± 4.6	46.6 ± 6.0	0.26 (SZ) ; 0.99(BD)
	Age at onset	NA	25.7 ± 8.1	24 ± 7.4, NA(2)^a^	
	Postmortem Interval (PMI)	24.3 ± 5.1	30.3 ± 5.3	27 ± 7.0	0.07 (SZ) ; 0.44 (BD)
	Race (w/h)	5/1	4/2	5/2	1.0 (SZ) ; 1.0 (BD)

*P*-values are calculated using t test and Fisher's exact test for continuous and categorical traits, respectively;

a Two patients did not record the age at onset. And the mean ± SD are calculated using data of remaining samples;

Race: w denotes for White, h denotes for Hispanic and n denotes for Native American;

NA, not available;

### Constructing lincRNA transcriptome in three brain regions of SZ and BD

We generated 562, 242 and 238 million reads for BA11, BA24 and BA9 samples, respectively, which were subsequently mapped to UCSC hg19 human genome. On average, there were 12.7 million reads per sample (Table S1), which was sufficient to detect differential genes [11]. To comprehensively characterize the lincRNA landscape in SZ and BD, we obtained 7952 known lincRNAs from GENCODE v19 and identified 168 novel lincRNAs using a customized computational pipeline based on all RNA-seq datasets from the three brain regions (Figure S1A, Tables S2–S3, see Supplementary Methods for details). After removing lowly expressed lincRNA genes and protein-coding genes (PCGs), we constructed the transcriptome for each brain region. We identified 1411 known lincRNAs, 91 novel lincRNAs and 14606 PCGs in BA11; 1412, 90 and 14741 in BA24; and 1461, 92 and 14798 in BA9.

We found that 73.3% of known lincRNAs, 87.6% of novel lincRNAs and 95.7% of PCGs expressed in all three brain regions, and 15.0% of known lincRNAs, 6.2% of novel lincRNAs and 1.9% of PCGs expressed in only one brain region (Figure S1B). The expression levels of known and novel lincRNAs were lower than PCGs in all three brain regions (Figure S1C). These lincRNAs had significantly lower coding potential and ORF ratio (ratio of ORF size to transcript length) relative to PCGs (*P*-value < 0.001, Wilcoxon rank sum test, Figures S1D-E) as measured by CPAT [12]. Moreover, the overall lengths of lincRNAs were significantly shorter than PCGs (*P*-value < 0.001, Wilcoxon rank sum test, Figure S1F). By calculating the exon sequence conservation using phastCons [13], we found that these lincRNAs were less conserved than PCGs (*P*-value < 0.001, Wilcoxon rank sum test, Figure S1G). These findings were in agreement with previous studies [14].

### Brain region-specific dysfunctional lincRNAs contribute to the pathogenesis of SZ and BD

**Figure 1:**
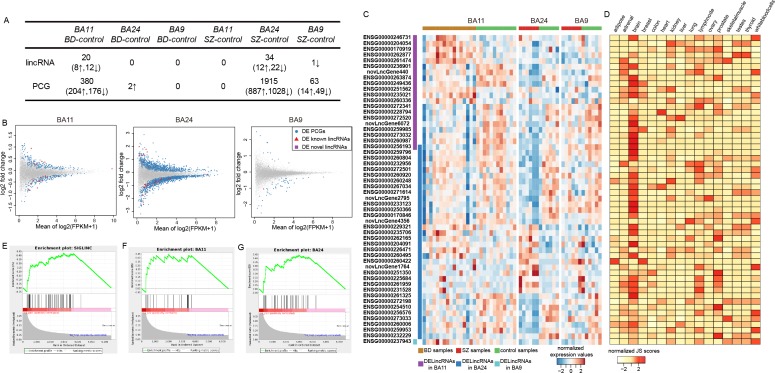
Differential analyses of lincRNAs in three brain regions for BD and SZ **A.** Statistics of differentially expressed lincRNAs and PCGs. **B.** MA-plots for comparisons between BD or SZ cases and controls in corresponding brain regions. Blue circles, red triangles and violet rectangles denote differentially expressed (DE) PCGs, known and novel lincRNAs, respectively. **C.** Heatmap representing normalized expression levels of differentially expressed lincRNAs (DELincRNAs) in three brain regions of corresponding disease state. **D.** Heatmap representing normalized JS scores of the DELincRNAs across 16 tissues. **E.**-**G.** GSEA plots for enrichment of brain-specific lincRNAs in all DELincRNAs (E), DELincRNAs in BA11_BD (F), and DELincRNAs in BA24_SZ (G).

We performed differential expression analyses in three brain regions by comparing SZ or BD cases to controls using DESeq2 [15], edgeR [16] and voom-limma [17] (see Materials and Methods section). An outstanding study had performed comprehensive evaluation of differential gene expression analysis methods for RNA-seq data. They demonstrated that DESeq and edgeR had good performance on detecting differential genes with high specificities and sensitivities, while voom-limma had favorable modeling of genes expressed in one condition (i.e. expressed only in cases or only in controls). Notably, all the three methods had good control of false positive errors [18]. Another study also highlighted that employing two or more differential analysis methods in parallel could enhance the overall sensitivity to detect true positive differential genes [19]. Moreover, by comparing the three sets of differential results, we found most of the differentially expressed genes were identified by more than one algorithm (Figure S2). Thus, to maximize identification of potential genes involved in BD or SZ, we combined the three algorithms to identify differentially expressed genes. As a result, for SZ, 34 differentially expressed lincRNAs (DELincRNAs) and 1915 differentially expressed PCGs (DEPCGs) were identified in BA24 (BA24_SZ), 1 and 63 in BA9 (BA9_SZ), and none in BA11 (Figure 1A-B). Specially, we did not identify any common DELincRNAs between BA24 and BA9. For BD, we identified 20 DELincRNAs and 380 DEPCGs only in BA11 (BA11_BD). When comparing SZ and BD, irrespective of brain regions, only one common DELincRNA between BA11_BD and BA24_SZ was revealed (Figure 1C), while the overlap was significant for DEPCGs (*P*-value < 0.001, Chi-square test). Such strong brain region specificity of dysfunctional lincRNAs indicated different roles of lincRNAs in different brain regions of major psychosis.

The tissue specificity of lincRNAs has been widely confirmed. To determine whether these brain region-specific DELincRNAs are brain tissue specific, we downloaded RNA-seq data from 16 normal tissues. Our results showed that these DELincRNAs displayed significant brain-specific patterns (Figure 1D-G), further supporting their critical roles in maintaining the homeostatic functions of the brain [20, 21].

By interrogating DEPCGs, we observed that numerous DEPCGs have been found to be associated with SZ and BD (*P*-value < 0.05, Chi-square test, Figure S3A). For example, SZ- and BD-related genes *BDNF* [22] and *GABRA1* [23] were dysregulated in both BA11_BD and BA24_SZ, and SZ-related gene *NPY* [24] was dysregulated in BA9_SZ. In addition, we observed significant overlaps (*P*-value < 0.05, Chi-square test, Figures S3B-C) between DEPCGs from our RNA-seq data and previous microarray data (GSE12649 and GSE53987 in GEO database [25] and three datasets in National Brain Databank, fold change>1.2). Through GO enrichment analysis of DEPCGs, we also identified some functions known to be involved in SZ pathology, such as synaptic transmission [26], central nervous system development [27] and oligodendrocyte differentiation [28] (Figure S4). These results provided validations for the differential expression of our RNA-seq data.

Taken together, our results showed that dysfunctional lincRNAs harbored strong brain and brain region specificity, suggesting that they could participate in important biological processes in the brain and contribute to the pathogenesis of SZ and BD.

### Dissecting dysfunctional lincRNA modules in major psychosis

In order to explore functional mechanisms of lincRNAs in major psychosis, we leveraged the “guilt-by-association” principle. We attempted to integrate individual expression differences of lincRNAs and PCGs into a higher order, systems level by applying the WGCNA approach [29, 30]. For BA24_SZ, BA9_SZ and BA11_BD, we identified 5, 4 and 3 significant co-expression modules (FDR < 0.05, permutation test, see Materials and Methods section) that contained at least one lincRNA and showed significant enrichment of DELincRNAs or DEPCGs (FDR < 0.05, hypergeometric test), respectively. These modules were regarded as dysfunctional lincRNA modules.

**Figure 2:**
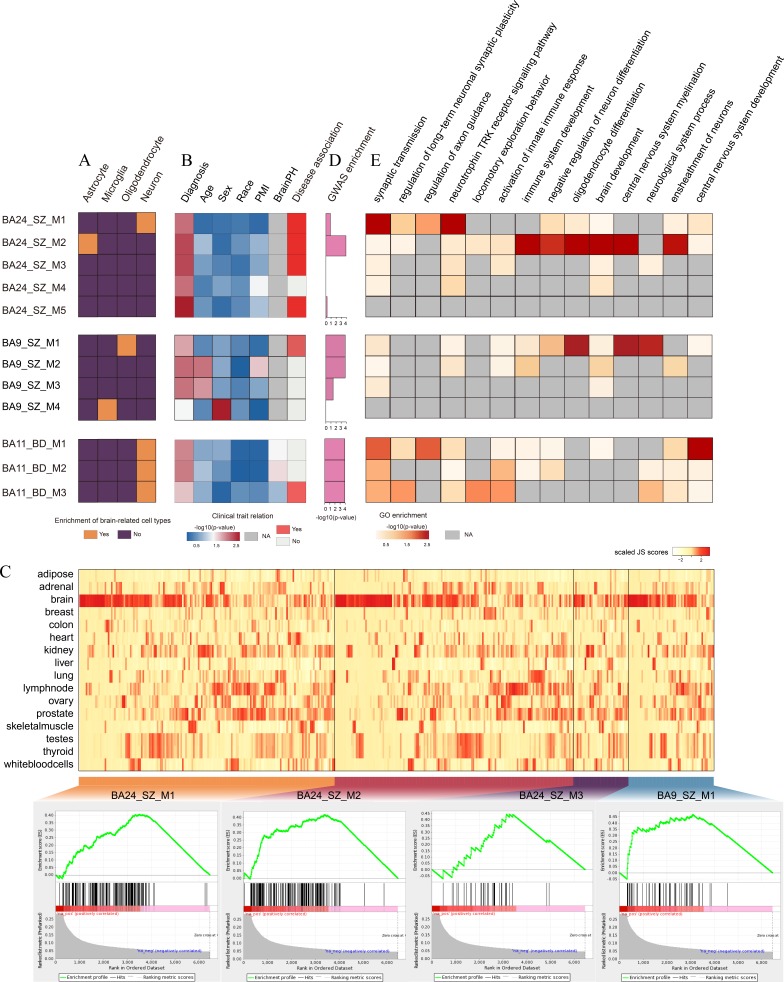
Co-expression network analysis of dysfunctional lincRNA modules **A.** Enrichment of brain-related cell types. Yellow grids indicate significant enrichment of the corresponding cell types in the corresponding modules. For each module, only the most significant cell type was given. **B.** Clinical trait relation. Heatmap representing -log10 transformed *P*-value of correlation test between module eigengenes and clinical traits. Red grids in disease association column represent significant association between the modules and corresponding disease while without significant correlation with other clinical traits. **C.** Enrichment of brain-specific lincRNAs in the dysfunctional modules. The upper panel shows the heatmap representing JS scores of lincRNAs and PCGs in the modules. The lower panel presents GSEA images indicating significant enrichment of brain-specific lincRNAs in the modules. **D.** Enrichment test of SZ- or BD-associated GWAS signals in the modules. The height of the bars denote -log10(*P*-value). **E.** Heatmap representing -log10 transformed *P*-value of functional enrichment analysis. Grey grids indicate NA values.

To explore the roles of the dysfunctional lincRNA modules, we estimated over-representation of brain-related cell types (Figure 2A, Supplementary Methods). We found that dysfunctional lincRNA modules detected in different brain regions were enriched for different cell type markers. M1 and M2 in BA24_SZ were enriched for neuronal and astrocyte markers, respectively. M1 and M4 in BA9_SZ were enriched for oligodendrocyte and microglia markers, respectively. Three modules in BA11_BD were all enriched for neuronal markers. Also, we examined the relationship between these modules and clinical traits including diagnosis, age, sex, race, PMI and brain PH. As a result, we found four modules (M1, M2, M3 and M5) in BA24_SZ, one module (M1) in BA9_SZ and one module (M3) in BA11_BD were associated with disease diagnosis without significant influences from confounding factors (Figure 2B). Among these disease-related modules, M1, M2 and M3 in BA24_SZ and M1 in BA9_SZ were significantly enriched by brain-specific lincRNAs (FDR < 0.25, GSEA, Figure 2C), highlighting the important roles of lincRNAs in the pathology of major psychosis. In order to further verify their potential disease contributions, we then performed GWAS enrichment analyses. As a result, M2 in BA24_SZ, M1 in BA9_SZ and M3 in BA11_BD (Tables S4–S6) showed significant enrichment of SZ- or BD-associated GWAS signals (Figure 2D).

We next investigated biological processes of the modules by performing functional enrichment analyses based on PCGs included in the modules (Figure 2E). We found that these dysfunctional lincRNA modules were involved in some important functions previously implicated in SZ and BD. For instance, M2 in BA24_SZ was involved in immune system development [31], negative regulation of neuron differentiation [32] and brain development [33]. M1 in BA9_SZ participated in oligodendrocyte differentiation [28] and central nervous system myelination [34]. M3 in BA11_BD was involved in synaptic transmission [35], locomotory exploration behavior [36] and activation of innate immune response [37]. These dysfunctional lincRNA modules detected in different brain regions seemed to be associated with different functions.

Taken together, our findings suggested that the dysfunctional lincRNA modules could contribute to psychiatric diseases through influencing different brain-related cell types in distinct brain regions, further supporting the important roles of lincRNAs in the pathology of SZ and BD.

### Abnormal epigenetic alterations of differentially expressed lincRNAs

**Figure 3:**
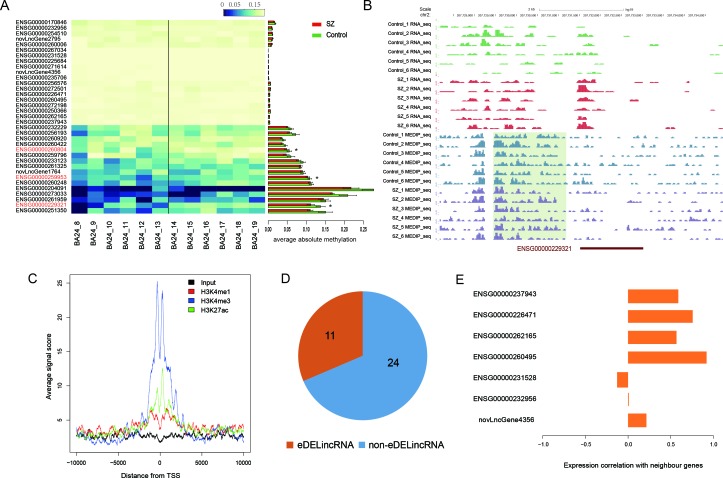
Epigenetic analysis of DELincRNAs in BA24 **A.** The heatmap of DNA methylation levels (left) and the corresponding ams (right) of promoters of DELincRNAs. **B.** The UCSC Browser screenshot showing the RNA-seq and MeDIP-seq signals within and around DELincRNA *ENSG00000229321*. **C.** Histone modification profiles within -10kb to +10kb from TSS of DElincRNAs. **D.** Pie plot showing the percentage of eDELincRNAs in all DELincRNAs. **E.** Barplots of expression correlations between 7 eDELincRNAs and their neighboring genes whose expression data were available.

DNA methylation is an important regulator of transcription and involved in many diseases. We thus explored whether the differential expression of lincRNAs was caused by DNA methylation alteration. To this end, we analyzed our previously detected MeDIP-seq data of 6 SZ patients and 6 controls. We found three DELincRNAs in BA24 and BA9 showing significant methylation differences (*P*-value < 0.05, Wilcoxon ranked sum test, Figure 3A). As an example, the promoter of DELincRNA *ENSG00000229321* showed lower methylation levels in SZ patients than in controls (Figure 3B). Consistently, we observed an obvious upregulation of *ENSG00000229321* expression in SZ patients compared with controls (*P*-value = 0.001, one-tailed Student's t-test). Interestingly, two of the three differentially methylated DELincRNAs were contained in the dysfunctional module M1 of BA24_SZ.

Previous studies have reported the presence of lincRNAs with enhancer-like function and their critical roles in development and differentiation [38]. To determine whether our identified DELincRNAs could function as enhancers, we examined the H3K4me3, H3K4me1 and H3K27ac signals around the promoters of these DELincRNAs in anterior cingulate cortex. As a result, we observed significant enrichment of H3K4me3 and H3K27ac signals at the promoters (Figure 3C). Subsequently, we identified 11 enhancer-associated DELincRNAs (eDELincRNAs) (Figure 3D, see Materials and Methods section), most of which were positively correlated with their closest PCGs in SZ (Figure 3E). Further, we found that these eDELincRNAs were involved in homotypic cell-cell adhesion and regulation of blood coagulation by functional analysis of their neighboring genes. Molecular pathways involved in neuronal cell adhesion were shown to contribute to SZ susceptibility [39]. A population-based cohort study revealed that SZ patients exhibited higher risk for developing deep vein thrombosis [40], formation of blood clots within deep veins. These results indicated that dysfunctional enhancer lincRNAs might play a potential role in the etiology of major psychosis.

## DISCUSSION

We performed RNA sequencing on three brain regions to systematically characterize potential roles of lincRNAs in SZ and BD. In BD, we identified DELincRNAs only in BA11. In SZ, the majority of DELincRNAs were identified in BA24. The two brain regions orbitofrontal cortex (BA11) and anterior cingulate (BA24) have been implicated in the pathology of BD [41] and SZ [42], respectively. Moreover, these DELincRNAs displayed strong brain-specific expression across different tissues. Such strong brain-region and brain specificity of DELincRNAs implied important roles of lincRNAs in the etiology of major psychosis, and were probably related to distinct functional roles of different brain regions in major psychosis. In comparison to BA24 in SZ, we identified few DELincRNAs (only one) in BA9. A possible explanation is that anterior cingulate (BA24) is more vulnerable than dorsolateral prefrontal cortex (BA9) in SZ [42]. In addition, we compared DELincRNAs between SZ and BD, and found only one common DELincRNA. In contrast, a significant overlap of DEPCGs between SZ and BD was observed, including some known common susceptible genes such as *BDNF* [22] and *GABRA1* [23]. Although similar pathogenesis between SZ and BD has been reported in previous studies, most of these observations were based on protein-coding genes [43]. Our lincRNA analysis results indicated that lincRNAs may exert different molecular functions between SZ and BD in a brain region-specific way.

Importantly, we revealed several dysfunctional lincRNA modules in different brain regions, and found that these modules could perform different functions in different brain regions. Specially, M2 in BA24_SZ, M1 in BA9_SZ and M3 in BA11_BD, which were associated with disease states and enriched for disease-related cell types and GWAS signals, were implicated in brain development, oligodendrocyte differentiation and locomotory exploration behavior, respectively. These three modules were likely to play important roles in the pathology of SZ or BD.

There were 11 dysregulated lincRNAs in the three dysfunctional lincRNA modules, including 9 in BA24_SZ, 1 in BA9_SZ and 1 in BA11_BD. In order to validate these lincRNAs, we investigated their expression changes in an external data set of 15 schizophrenia, 18 bipolar disorder and 18 control samples based on microarray profiling (GSE53987). We re-annotated the expression of 8 out of the 11 dysregulated lincRNAs among which 7 (87.5%) were dysregulated with the same direction as in our results (Figure S5). The schemes of the structures of dysregulated lincRNAs in the three dysfunctional lincRNA modules were provided in Figure S6. To explore the potential regulation mechanism of DELincRNAs on their neighboring genes, we investigated the Hi-C data in human neuronal precursor cells [44]. As a result, we observed strong chromatin interactions between most of the dysregulated lincRNAs and promoter regions of their neighboring genes (Figure S7). These results suggested that lincRNAs could form chromosome loop with their neighboring genes to exert regulation functions.

Among the 151 lincRNAs in M2 in BA24_SZ, 9 showed differential expression. Functional characterization of individual lincRNAs using their highly co-expressed PCGs (PCC>0.8) in the module revealed that some lincRNAs were associated with ensheathment of neurons, myelination and metabolic processes et al. (Figure S8A). We noted that DELincRNA *ENSG00000260495* was presented in this module, which showed strong brain specificity (JS score = 0.42), implying a possible origin of brain tissue. Interrogating PCGs highly co-expressed with *ENSG00000260495*, we found that although most of them located in different chromosomes there were still several PCGs locating in the same chromosome with *ENSG00000260495* (Figure S9F). Moreover, *ENSG00000260495* was identified as an eDELincRNA, and its expression was positively correlated with that of its neighboring gene GAN, suggesting a cis-regulation mechanism. Interestingly, strong chromatin interaction was observed between *ENSG00000260495* and the promoter region of GAN when investigating Hi-C data in human neuronal precursor cells (Figure S7F). The structural relation between them provided support for the cis-regulation of *ENSG00000260495* on *GAN*. *GAN* has been demonstrated to play a role in neurofilament architecture [45] and its instability could cause giant axonal neuropathy [46]. Neurofilament subunits NF-L and NF-M played crucial roles in sustaining the neuronal cytoskeleton and both of them were reported to be increased in SZ [47]. Our work provided new targets to explore the potential roles of lincRNAs in the pathogenesis of major psychosis.

*ENSG00000237943* (*PRKCQ-AS1*) was the DELincRNA in M1 in BA9_SZ involved in oligodendrocyte differentiation and central nervous system myelination (Figure S8B). The functional category oligodendrocyte differentiation contained five DEPCGs of SZ, including *CNP, MYRF, FA2H, CNTN2* and *PLP1. CNP* and *QKI*, two candidate genes for schizophrenia, have been reported to be down-regulated in the schizophrenic brain [48, 49]. Although the majority of the PCGs highly co-expressed with *ENSG00000237943* located in different chromosomes (Figure S9J), *ENSG00000237943* was still proved to be an eDELincRNA in our results and transcribed divergently within 1kb of the promoter of *PRKCQ*. Moreover, the expression level of *ENSG00000237943* in BA9 was highly positively correlated with that of PRKCQ (PCC = 0.83) and there was strong chromatin interaction between *ENSG00000237943* and the promoter region of *PRKCQ* (Figure S7J). *PRKCQ* has been identified as a positive regulator in diverse cellular signaling pathways and diseases, such as T-cell activation and central nervous system autoimmune disease [50, 51]. Interestingly, *PRKCQ* is also involved in the differentiation of oligodendrocytes [52]. These data suggested that the *ENSG00000237943* dysregulation could induce oligodendrocyte dysfunction by trans-regulating DEPCGs in BA9_SZ_M1 or cis-regulating PRKCQ in SZ.

M3 in BA11_BD contained 9 lincRNAs and 331 PCGs, among which 1 lincRNA (*ENSG00000228794*) and 19 PCGs were differentially expressed. Notably, the DELincRNA *ENSG00000228794* was located in 1p36.33, a region linked with BD [53]. Moreover, functional characterization through highly co-expressed PCGs (Figure S9K) in the module demonstrated that *ENSG00000228794* could be involved in calcium ion transport (Figure S8C), a critical component of calcium signaling. Notably, calcium signaling was found to play important roles in modulation of synaptic plasticity [54] and was shown to be disturbed in BD [55]. Thus, it's reasonable to assume that *ENSG00000228794* could contribute to pathogenesis of BD and further characterization would help better understanding molecular mechanisms of BD.

Previous studies have performed comparative gene expression analysis between brain tissues and peripheral blood samples of schizophrenia patients and demonstrated concurrent up-regulation of SELENBP1 which was proposed as a candidate biomarker for SZ [56]. A meta-analysis of peripheral BDNF levels in adults with BD revealed consistent reduction of BDNF levels and suggested peripheral BDNF as a biomarker of mood states and disease progression for BD [57]. These findings suggest the potential clinical value of investigating gene expression levels in peripheral blood samples of major psychosis. Thus, future studies should be focused on investigating the dysfunctional lincRNAs in peripheral blood samples of major psychosis to identify potential lincRNA biomarkers. These candidate lincRNA biomarkers could be used to help clinically guide the diagnosis and treatment of major psychosis.

In summary, we identified a number of dysregulated lincRNAs in SZ and BD, and found their distinct contributions in different brain regions. Our findings revealed essential functions of lincRNAs in psychiatric disorders, helped us understand the molecular mechanisms of SZ and BD and further investigation of lincRNAs in peripheral blood samples of SZ and BD in the future could help identify diagnostic markers.

## MATERIALS AND METHODS

### Subjects

We obtained 82 post-mortem brain tissues from BA11, BA24 and BA9 to conduct RNA sequencing. BA11 samples from 16 SZ cases, 16 BD cases and 12 psychiatrically normal controls were obtained from the Array Collection in Stanley Medical Research Institute (SMRI) [58] and ethics approval for brain collection was held by the SMRI. Tissues from BA24 and BA9 of 6 SZ cases, 7 BD cases and 6 controls were retrieved from the Southwest Brain Bank [59] with ethics approval from the Institutional Review Board of Texas Tech University Health Science Center.

### RNA-seq analysis

RNA extraction and sequencing were carried out as previously described [59]. Briefly, total RNA was collected from brain tissues, which was then used to isolate poly(A) mRNA. The mRNA was fragmented to 200~300bp in size and reverse-transcribed to cDNA, followed by purification, ligation of sequencing adapters and amplification with PCR. Libraries were sequenced on Illumina HiSeq 2000 to generate paired-end 100 bp sequence reads. Raw read data were deposited in NCBI Gene Expression Omnibus (GEO) under accession number GSE78936.

RNA-seq reads were mapped to the human reference genome (version hg19) using Tophat (version 2.0.13) [60] with default parameters. Only uniquely mapped reads with no more than two mismatches were retained. Read counts for each lincRNA and protein-coding gene (PCG) were computed using HTSeq [61]. For each brain region, lowly expressed genes (read count < 2 in more than 50% of samples) were removed. Additionally, fragments per kilobase of transcript per million fragments mapped (FPKM) value was calculated for each lincRNA and PCG.

### Differential expression analysis

Differential expression between cases (SZ or BD) and controls was assessed using three methods including DESeq2 [15], edgeR [16] and voom-limma [17]. For each brain region, genes with an adjusted *P*-value of < 0.05 in at least one method were considered as differentially expressed genes.

### Functional annotation

We performed Gene Ontology [62] enrichment analysis using the hypergeometric distribution test with FDR < 0.05, which was implemented in the package GOstats [63].

### Tissue specificity of lincRNAs

FPKM values of lincRNA genes in 16 normal tissues were estimated from RNA-seq data of the Human Body Map 2.0. To evaluate the tissue specificity of a lincRNA, we calculated the Jensen-Shannon tissue specificity score (JS score) for each tissue based on an entropy-based metric used by Cabili et al. [64]. A lincRNA was regarded to be tissue-specific if the maximal JS score across all tissues was over 0.4.

### Weighted gene co-expression network analysis

We utilized Weighted Gene Co-expression Network Analysis (WGCNA) [29] to identify co-expression modules based on lincRNA and PCG expression profiles. Permutation test was used to calculate significance (FDR < 0.05). The modules with enrichment of differentially expressed lincRNAs or PCGs (hypergeometric test, FDR < 0.05) and containing at least one lincRNA were determined as dysfunctional lincRNA modules. To further explore the roles of dysfunctional lincRNA modules, we then inspected whether the dysfunctional lincRNA modules showed enrichment of brain-related cell types, association with disease states and enrichment of SZ- or BD-associated GWAS signals [65, 66]. Based on PCGs in each module, GO enrichment analysis was performed to predict biological functions for the module. See Supplementary Methods for details.

### Epigenetic analysis

Methylated DNA immunoprecipitation and sequencing (MeDIP-seq) of 6 SZ patients and 6 controls was performed as previously described [59]. We utilized MEDIPS package (version 1.12.0) [67] to quantify the methylation levels at the promoters of lincRNAs (2 kb upstream and 0.5kb downstream of transcriptional start sites). Differential methylation of the DELincRNAs were assessed using Wilcoxon rank sum test with *P*-value < 0.05. ChIP-seq data for histone marks H3K4me1, H3K4me3 and H3K27ac of BA24 were obtained from the NIH Roadmap Epigenomics Program [68] (GSE17312). A lincRNA was defined as an enhancer-associated lincRNA if its promoter was significantly enriched for H3K27ac peaks identified by MACS [69] (*P*-value < 10^−5^ with default parameters). See Supplementary Methods for details.

### Statistical analysis

All statistical analyses were performed with R software. For comparisons of clinical traits between cases and control samples, t-test and Fisher's exact test were performed for continuous and categorical traits, respectively. Wilcoxon rank sum test was conducted for comparisons between characteristics of lincRNAs and protein-coding genes. To calculate the statistical significance of overlap between DEPCGs in BA11_BD and BA24_SZ and overlap between DEPCGs of this study and disease-associated genes from GAD database and between DEPCGs of this study and that of previous microarray studies, Chi-square test was carried out. For enrichment of DEPCGs and DELincRNAs in modules, hypergeometric test was performed to assess the statistical significance.

## SUPPLEMENTARY MATERIALS FIGURES AND TABLES














