# Supplementary material for: Systematically characterizing dysfunctional long intergenic non-coding RNAs in multiple brain regions of major psychosis
Source: Oncotarget. 2016 Sep 19;7(44):71087–98. doi: 10.18632/oncotarget.12122 (PMC5342065; doi:10.18632/oncotarget.12122)
Supplement: Supplementary file 1 [file oncotarget-07-71087-s001.pdf]

Systematically characterizing dysfunctional long intergenic non-coding RNAs in multiple brain regions of major psychosis

Supplementary Material

Identification of novel lincRNAs

To identify novel lincRNAs, RNA-seq reads were first mapped to the human reference genome version hg19 using Tophat (version 2.0.13)[1] with default parameters. Only uniquely mapped reads were retained. Cufflinks (version 2.2.1)[2] was used to assemble aligned reads into transcripts, based on which we developed a method to identify novel lincRNAs. In details, we retrieved unannotated transcripts and mapped their exons (termed query exons) to the human reference genome to calculate the exon coverage of each base. In order to obtain a unified exon set, we defined two parameters including ceiling coverage and floor coverage, representing the upper and lower limits of exon coverage, respectively. Initially, putative exons were identified as genomic regions with exon coverages of all bases in them no less than the given ceiling coverage value. Then we compared each putative exon with query exons. If a putative exon satisfied three criteria: 1) the overlap with a query exon must account for ≥ 80% of the query exon; 2) the overlap with a query exon must account for ≥ 50% of the putative exon; 3) the number of query exons that satisfy both 1) and 2) must be more than the given ceiling coverage value, the putative exon was retained as a component of the unified exon set. Through iterative searches of putative exons with different coverage (ranged from ceiling coverage to floor coverage), a final unified exon set was constructed. Different values of ceiling coverage and floor coverage can be used to identify unified exons with different resolutions. In this study, both ceiling coverage and floor coverage were set to 8. Subsequently, unified exons were assembled into transcripts and we considered the ones with length ≥ 200bp and coding potential score (calculated by CPC tool) ≤ 0 as novel lncRNAs.

Weighted gene co-expression network analysis (WGCNA)

Network construction and module detection

Unsupervised co-expression network analysis was performed to identify modules of co-expressed genes using Weighted Gene Co-expression Network Analysis (WGCNA) package in R[3]. Since

we detected significantly differentially expressed lincRNAs in three contexts - comparisons between SZ cases and controls in BA24 (BA24_SZ for short) and BA9 (BA9_SZ for short) and comparisons between BD cases and controls in BA11 (BA11_BD for short), we constructed co-expression networks for these contexts. For each context, the FPKM values of filtered lincRNAs and PCGs (read count>2 in more than 50% samples) were normalized by log2 transformation (i.e. log2(FPKM+1)) and quantile normalized, then Pearson correlation coefficients were calculated for all gene pairs. The resulting correlation matrix was transformed to an adjacency matrix using a power function. A scale-free topology criterion[4] was adopted to choose the power (10, 9 and 5 for BA24_SZ, BA9_SZ and BA11_BD, respectively). Based on the adjacency matrix, topological overlap (TO, indicating relative interconnectedness between two genes) was calculated for each pair of genes. Then, modules were detected using average linkage hierarchical clustering based on the TO dissimilarity (1-TO). The minimum module size was set at 30 genes and the minimum height for merging modules at 0.25 for BA24_SZ and BA9_SZ, while minimum height of 0.15 was chosen for BA11_BD to obtain moderately large and distinct modules.

Identification of significantly co-expressed modules

To verify genes in the modules were co-expressed beyond by chance, we performed permutation test based on the assumption that the mean TO of a network module should be greater than that of a random module[5]. For each module, we randomly selected 100 sets of genes with equivalent size of the module from the network. The p-value was estimated as the ratio of random gene sets whose mean TO was greater than that of the module. Then, multiple comparison correction was performed for all modules in a network. Finally, modules with FDR<0.05 were considered significantly co-expressed modules.

Module characterization

Enrichment of DELincRNAs or DEPCGs in the significantly co-expressed modules were assessed using hypergeometric test with FDR<0.05. The modules which contained lincRNAs and were enriched with DELincRNAs or DEPCGs were retained for subsequent analyses. Over-representation of brain-related markers were performed using the WGCNA function userListEnrichment with Bonferroni corrected p-value<0.05. Correlation test between module eigengenes (equivalent to the first principal component[6]) and clinical traits such as diagnosis,

age, sex, race, PMI and brain PH were performed using corPvalueStudent function. Disease-associated modules were identified as those showing significant correlation (p-value<0.05) with disease state while non-significant correlation with other clinical traits.

To identify potentially disease-causal modules, we utilized the method described in[7] to perform gene-set-based GWAS enrichment analysis. Summary results of association of SNPs with SZ or BD were retrieved from[8, 9], respectively. For each gene in a given module, we assigned SNPs within 110 kb upstream and 40 kb downstream to it. We counted the number of SNPs (N) assigned to all genes in each module. Then we determined the proportion of SNPs with nominal association p-values<0.05 in the module. Next, we randomly selected N SNPs from GWAS data with replacement and calculated the proportion of SNPs with GWAS p-value<0.05. We repeated random selections 10000 times and the p-value of GWAS enrichment in a module was determined as the ratio of random selections whose proportion of nominally significant SNPs was greater than that of the given module. Finally, FDR correction was performed to identify modules with significant SZ- or BD-associated GWAS signal enrichment.

Epigenetic analysis

MeDIP-seq analysis

Methylated DNA immunoprecipitation and sequencing (MeDIP-seq) of 6 SZ patients and 6 controls was performed as previously described[10]. Briefly, DNA was collected from BA24 of samples and then fragmented by sonication to a mean size of approximately 250 bp, followed by end-blunting, dA addition to the 3'-end and ligation of adapters. After PCR amplification, DNA fragments were subjected to 50 bp paired-end sequencing using Illumina HiSeq2000. After quality control, raw reads were aligned to human reference genome hg19 using bowtie (version 1.1.1)[11] with two mismatches. Only the nonredundant, uniquely mapped reads were retained for subsequent analysis.

To quantify the methylation levels of lincRNA, MeDIP-seq data of BA24 was processed using MEDIPS package (version 1.12.0)[12]. Specially, the relative methylation score (rms) was calculated with a sliding window of 250 bp. Then the absolute methylation score (ams) which was corrected for CpG density was calculated for each DELincRNA in each sample through dividing mean rms by mean coupling factors of bins that located in the promoter (-2 to 0.5 kb from TSS).

Differential methylation was calculated using Wilcoxon rank sum test among 35 DELincRNAs. A lincRNA was defined as significantly differentially methylated if *P*-value<0.05.

ChIP-seq analysis

ChIP-seq data for histone marks H3K4me1, H3K4me3 and H3K27ac of BA24 were obtained from the NIH Roadmap Epigenomics Program[13] (GSE17312). A lincRNA was defined as an enhancer-associated lincRNA (elincRNA) if its promoter was significantly enriched for H3K27ac peaks calculated by MACS[14] (*P*-value<0.05).

Supplementary References

1. Trapnell C, Pachter L and Salzberg SL. TopHat: discovering splice junctions with RNA-Seq. Bioinformatics. 2009; 25(9):1105-1111.
2. Trapnell C, Williams BA, Pertea G, Mortazavi A, Kwan G, van Baren MJ, Salzberg SL, Wold BJ and Pachter L. Transcript assembly and quantification by RNA-Seq reveals unannotated transcripts and isoform switching during cell differentiation. Nature biotechnology. 2010; 28(5):511-515.
3. Langfelder P and Horvath S. WGCNA: an R package for weighted correlation network analysis. BMC bioinformatics. 2008; 9:559.
4. Zhang B and Horvath S. A general framework for weighted gene co-expression network analysis. Statistical applications in genetics and molecular biology. 2005; 4:Article17.
5. Oldham MC, Konopka G, Iwamoto K, Langfelder P, Kato T, Horvath S and Geschwind DH. Functional organization of the transcriptome in human brain. Nature neuroscience. 2008; 11(11):1271-1282.
6. Langfelder P and Horvath S. Eigengene networks for studying the relationships between co-expression modules. BMC systems biology. 2007; 1:54.
7. Akula N, Barb J, Jiang X, Wendland JR, Choi KH, Sen SK, Hou L, Chen DT, Laje G, Johnson K, Lipska BK, Kleinman JE, Corrada-Bravo H, Detera-Wadleigh S, Munson PJ and McMahon FJ. RNA-sequencing of the brain transcriptome implicates dysregulation of neuroplasticity, circadian rhythms and GTPase binding in bipolar disorder. Molecular psychiatry. 2014; 19(11):1179-1185.
8. Schizophrenia Working Group of the Psychiatric Genomics C. Biological insights from 108 schizophrenia-associated genetic loci. Nature. 2014; 511(7510):421-427.
9. Psychiatric GCBDWG. Large-scale genome-wide association analysis of bipolar disorder identifies a new susceptibility locus near ODZ4. Nature genetics. 2011; 43(10):977-983.
10. Xiao Y, Camarillo C, Ping Y, Arana TB, Zhao H, Thompson PM, Xu C, Su BB, Fan H, Ordonez J, Wang L, Mao C, Zhang Y, Cruz D, Escamilla MA, Li X, et al. The DNA methylome and transcriptome of different brain regions in schizophrenia and bipolar disorder. PloS one. 2014; 9(4):e95875.
11. Langmead B, Trapnell C, Pop M and Salzberg SL. Ultrafast and memory-efficient alignment of short DNA sequences to the human genome. Genome biology. 2009; 10(3):R25.
12. Chavez L, Jozefczuk J, Grimm C, Dietrich J, Timmermann B, Lehrach H, Herwig R and Adjaye J. Computational analysis of genome-wide DNA methylation during the differentiation of human embryonic stem cells along the endodermal lineage. Genome research. 2010; 20(10):1441-1450.

13. Bernstein BE, Stamatoyannopoulos JA, Costello JF, Ren B, Milosavljevic A, Meissner A, Kellis M, Marra MA, Beaudet AL, Ecker JR, Farnham PJ, Hirst M, Lander ES, Mikkelsen TS and Thomson JA. The NIH Roadmap Epigenomics Mapping Consortium. Nature biotechnology. 2010; 28(10):1045-1048.
14. Zhang Y, Liu T, Meyer CA, Eeckhoute J, Johnson DS, Bernstein BE, Nusbaum C, Myers RM, Brown M, Li W and Liu XS. Model-based analysis of ChIP-Seq (MACS). Genome biology. 2008; 9(9):R137.

Supplementary Figures

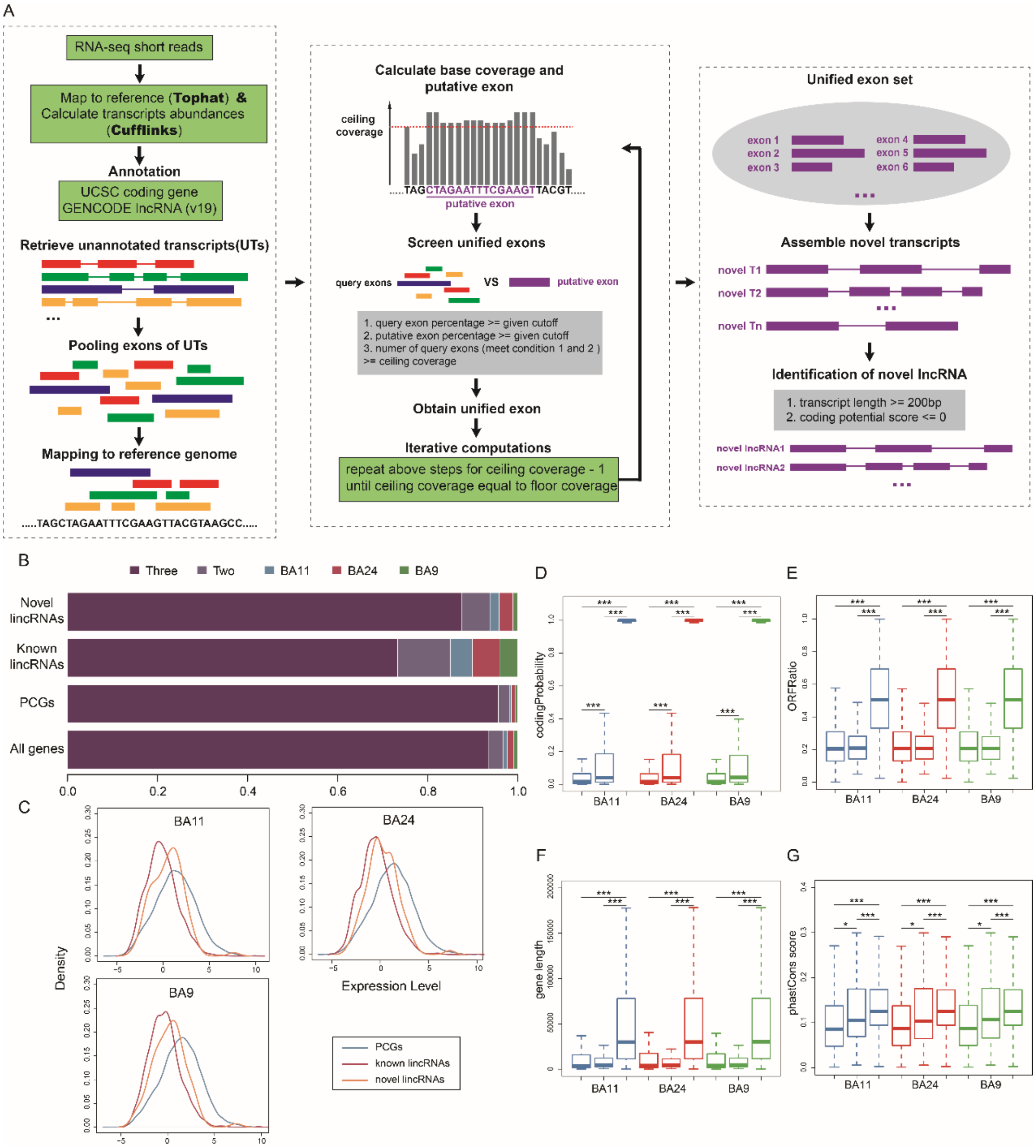

Supplementary Figure S1. Identification and characterization of lincRNA transcriptome. (A) Computational pipeline to identify novel lincRNAs. (B) Percentages of genes (including known lincRNAs, novel lincRNAs, PCGs and all genes) commonly expressed in three (purple) and two (light purple) brain regions, and specifically expressed in BA11 (blue), BA24 (red) and BA9 (green), respectively. (C) Distribution of expression levels for known lincRNAs, novel lincRNAs, and PCGs. (D) CPAT coding probability scores, (E) ORF ratios, (F) gene lengths and (G) conservation scores for known lincRNAs (left), novel lincRNAs (middle) and PCGs (right) in BA11, BA24 and BA9, respectively. Comparisons among the three gene sets were performed by Chi-square test. Asterisk (*), double asterisks (**) and triple asterisks (***) represent statistically significant differences at *p-value*<*0.05*, <*0.01* and <*0.001*, respectively.

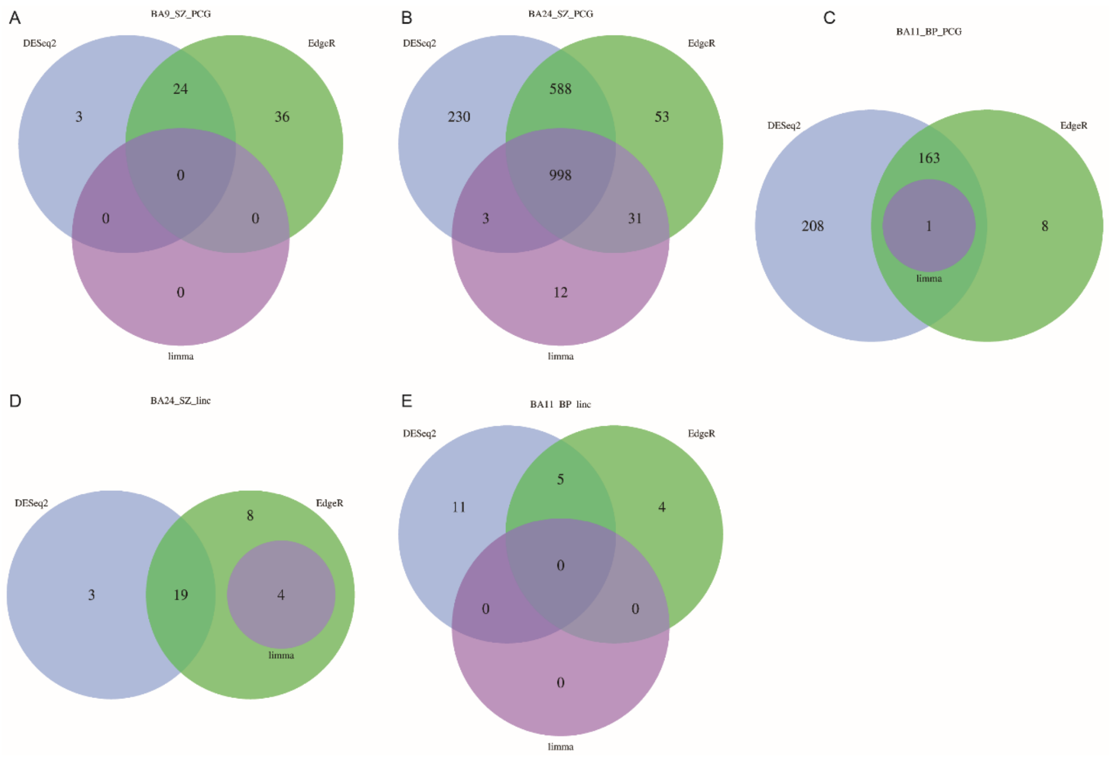

Supplementary Figure S2. Overlap between differential results of three algorithms (DESeq2, edgeR and voom-limma) when comparing differential protein-coding genes between schizophrenia and control in BA9 (A), comparing DEPCGs between SZ and control in BA24 (B), comparing DEPCGs between bipolar disorder (BD) and control in BA11 (C), comparing differential lincRNAs (DELincRNAs) between SZ and control in BA24 (D) and comparing DELincRNAs between BD and control in BA11 (E).

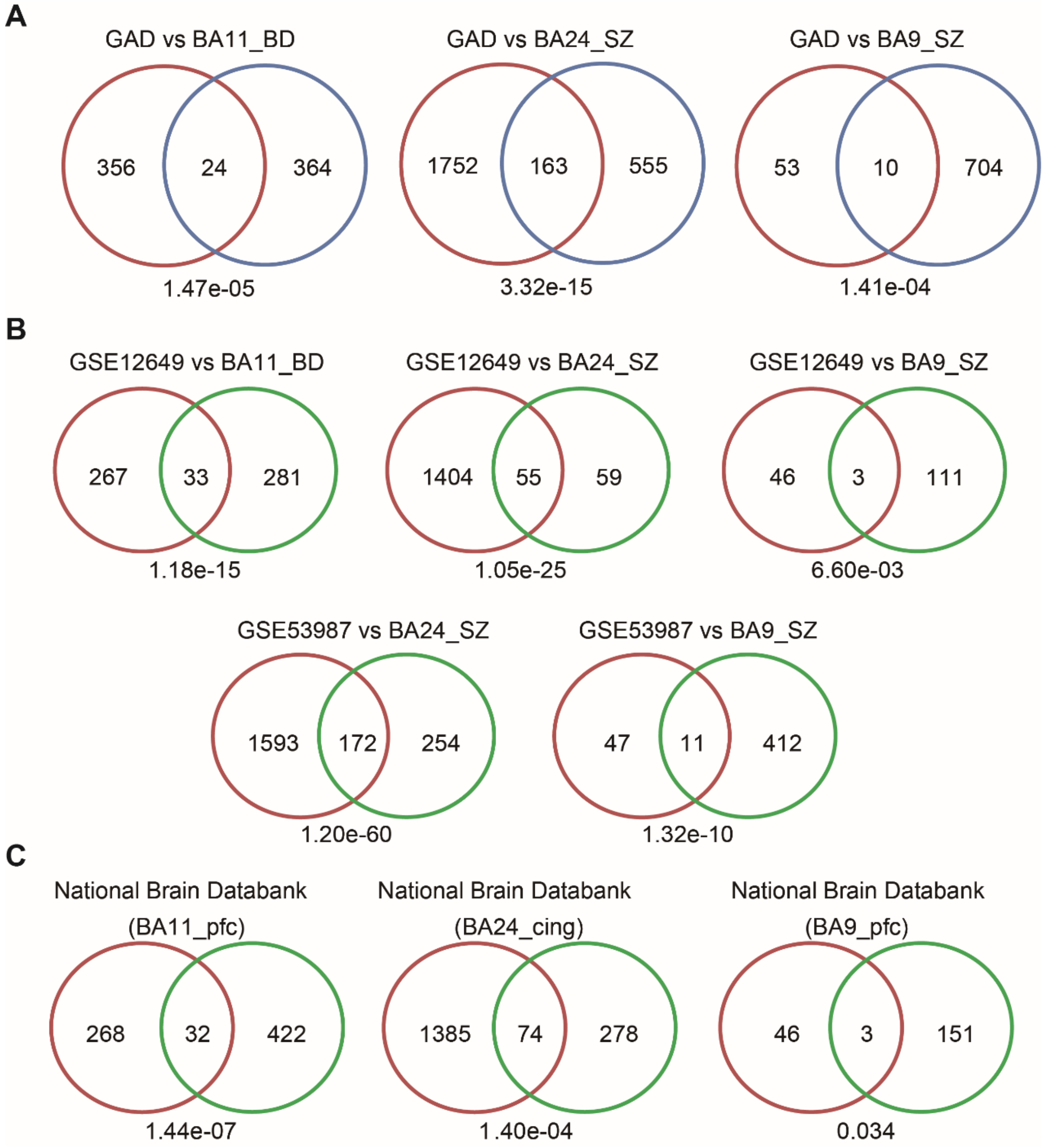

Supplementary Figure S3. Validation of our differential analyses results. (A) Overlaps between differentially expressed PCGs of BD in BA11 (left), SZ in BA24 (middle) and BA9 (right), and BD- or SZ-related PCGs in GAD database, respectively. (B, C) Overlaps between differentially expressed PCGs in our RNA-seq data and microarray data in the corresponding conditions obtained from GEO (B) and from National Brain DataBank (C). Comparisons between two gene sets were performed using Chi-square test.

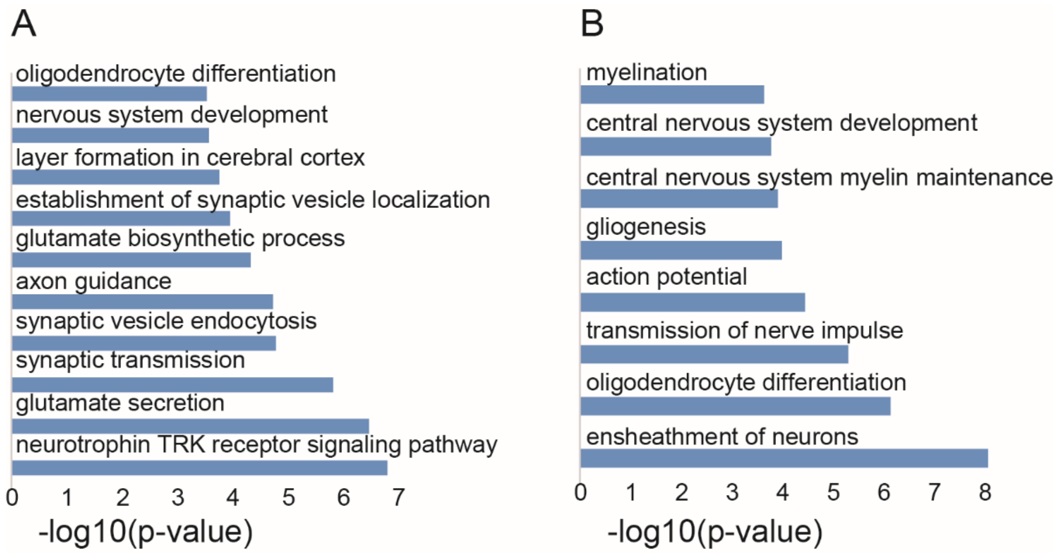

Supplementary Figure S4. GO enrichment analyses of DEPCGs in BA24_SZ (A) and BA9_SZ (B). There are no GO terms enriched by DEPCGs in BA11_BD.

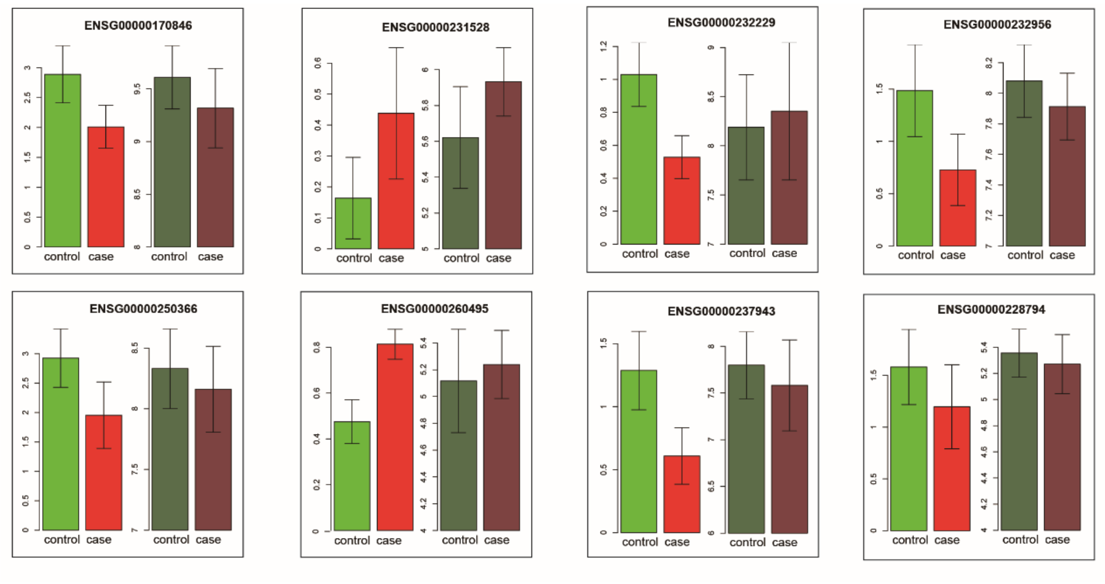

Supplementary Figure S5. Validation of dysregulated lincRNAs in the three dysfunctional lincRNA modules. The two left light-colored bars showed expression levels in our study, and two right dark-colored bars presented re-annotated expression levels in the microarray profiling data set (GSE53987). Green bars denoted expression levels in control samples and red bars in cases.

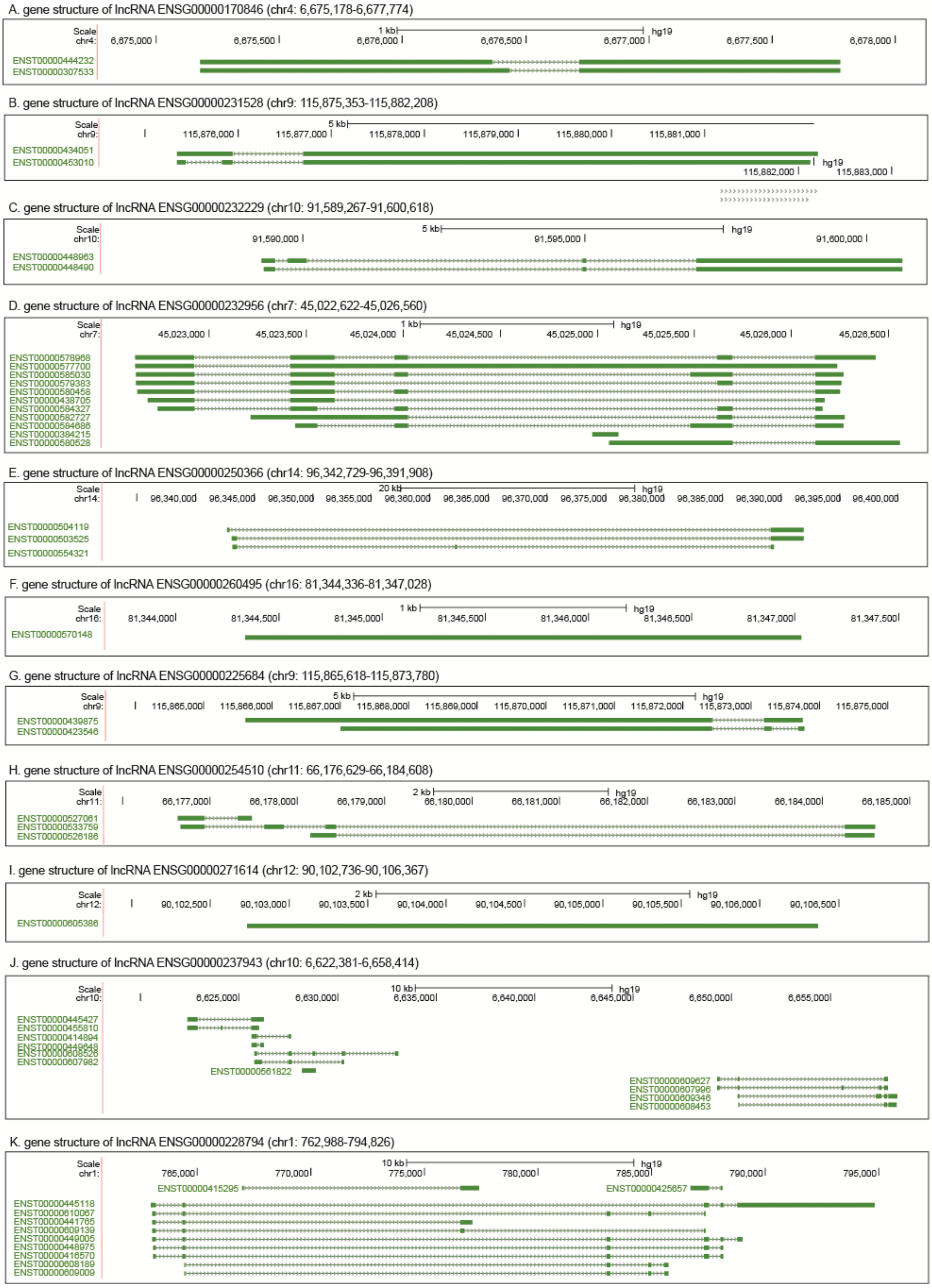

Supplementary Figure S6. Schemes of the structures of dysregulated lincRNAs in the three dysfunctional lincRNA modules.

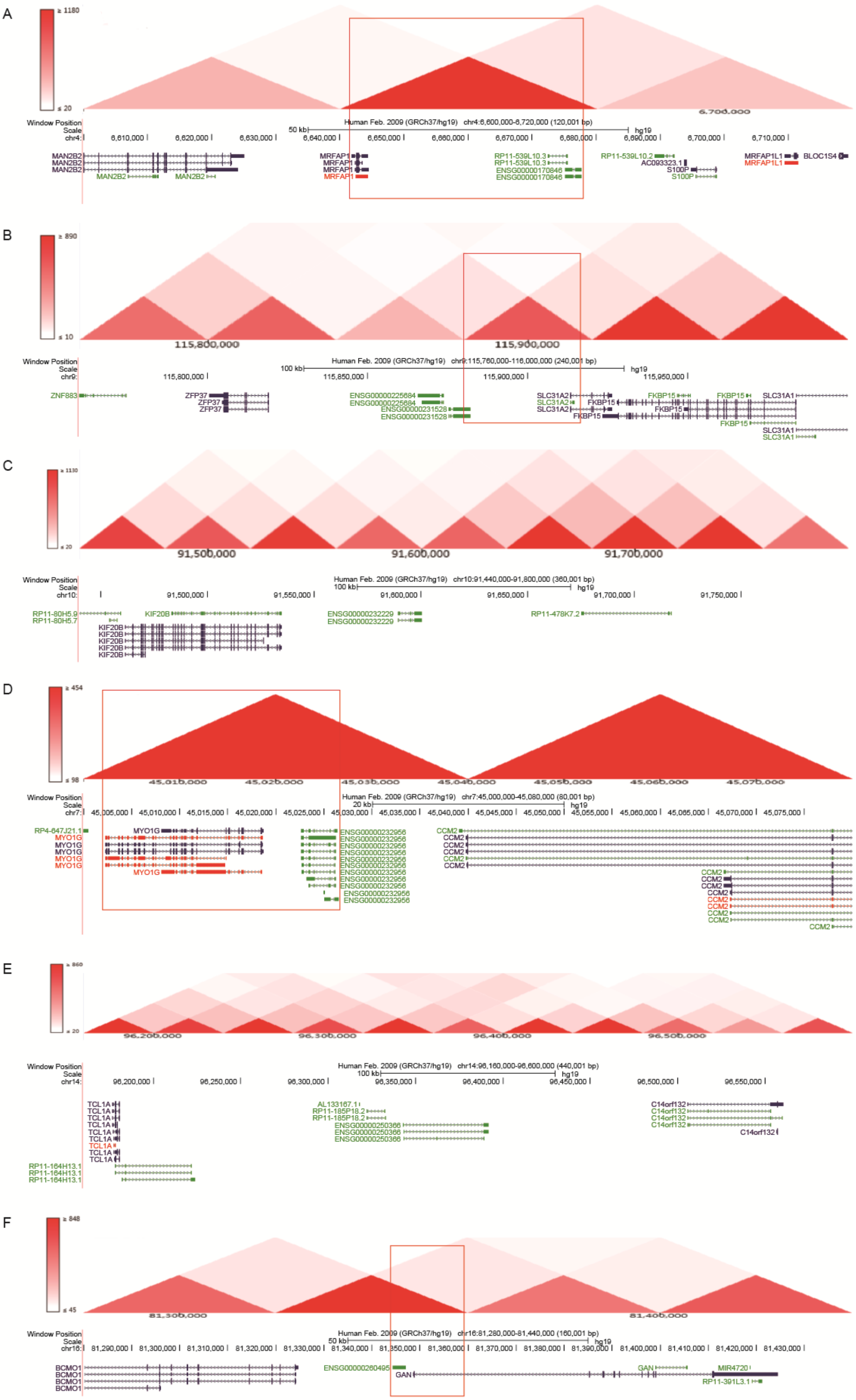

A
Human Feb. 2009 (GRCh37/hg19) chr4:6,600,000-6,720,000 (120,001 bp)
B
115,800,000
115,900,000
Human Feb. 2009 (GRCh37/hg19) chr9:115,760,000-116,000,000 (240,001 bp)
C
91,500,000
91,600,000
91,700,000
Human Feb. 2009 (GRCh37/hg19) chr10:91,440,000-91,800,000 (360,001 bp)
D
Human Feb. 2009 (GRCh37/hg19) chr7:45,000,000-45,080,000 (80,001 bp)
E
Human Feb. 2009 (GRCh37/hg19) chr14:96,160,000-96,600,000 (440,001 bp)
F
Human Feb. 2009 (GRCh37/hg19) chr16:81,280,000-81,440,000 (160,001 bp)

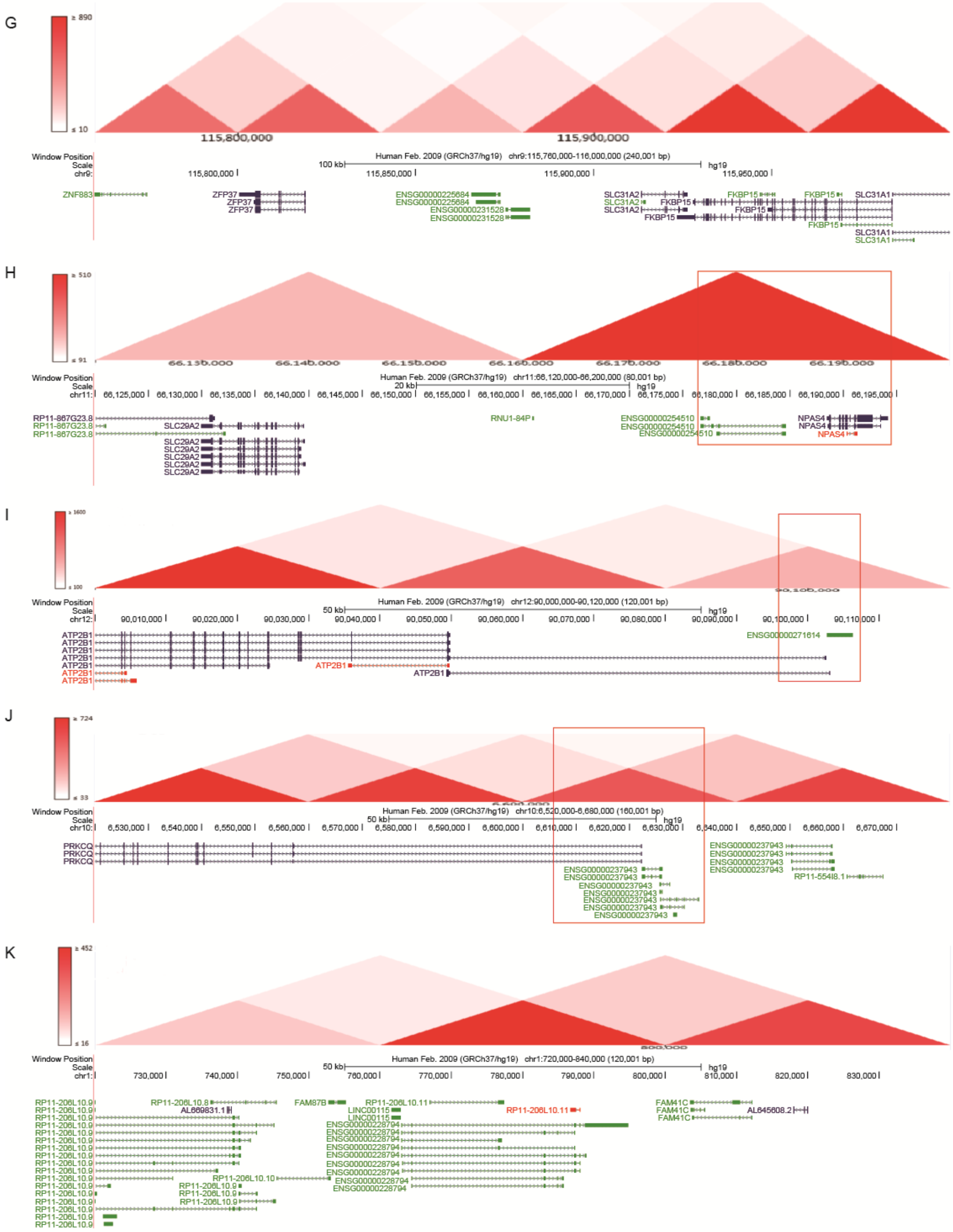

Supplementary Figure S7. Two-dimensional heatmap shows the Hi-C interaction frequency surrounding lincRNAs and their neighboring genes in human neuronal precursor cells. Strong interactions between lincRNAs and their neighboring genes were highlighted in red rectangles.

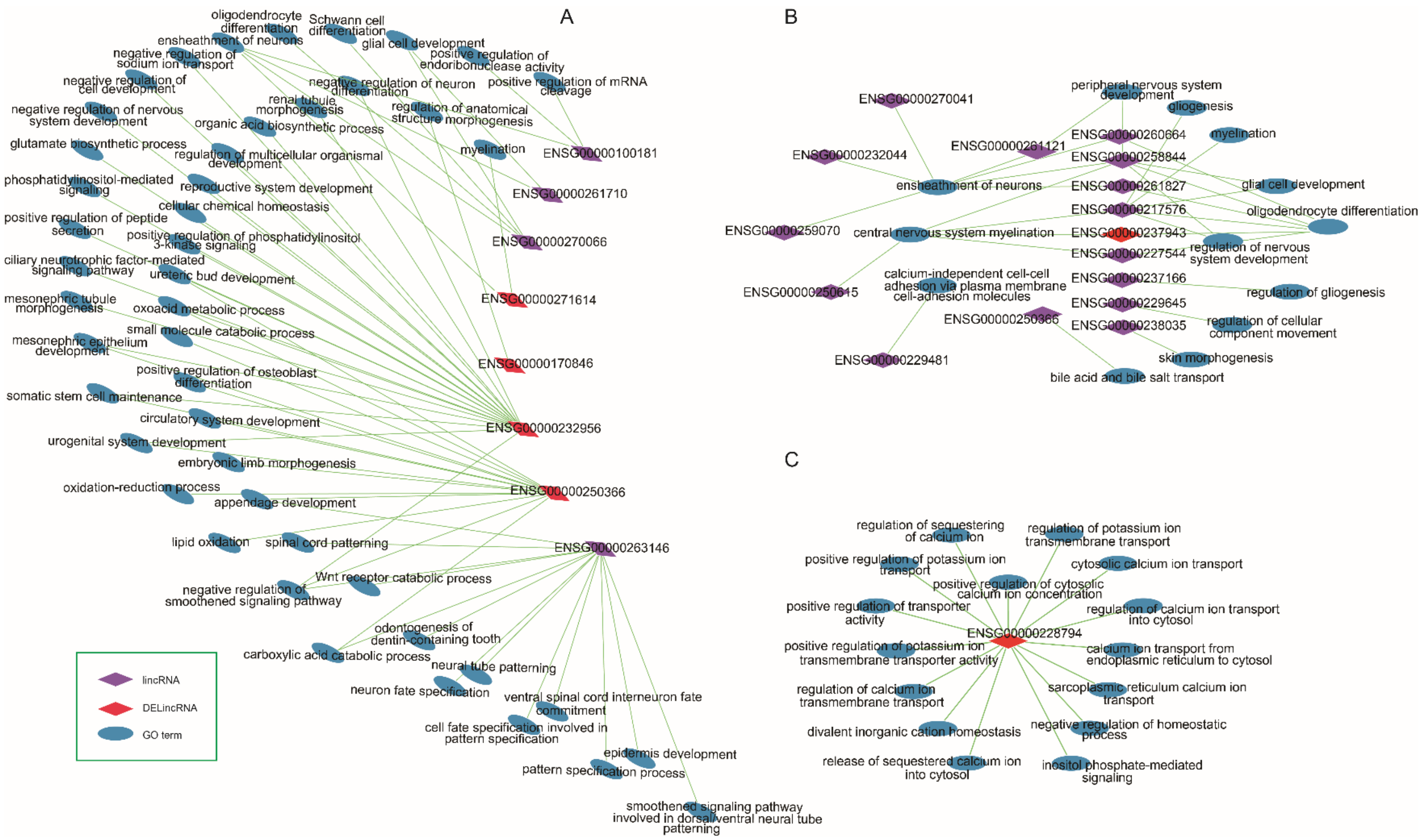

Supplementary Figure S8. Functional characterization of lincRNAs in the three most disease-related modules: BA24_SZ_M2 (A), BA9_SZ_M1 (B) and BA11_BD_M3 (C). Diamond nodes denote lincRNAs and red ones denote differentially expressed lincRNAs (DELincRNAs). To characterize functions for each lincRNA, we performed GO enrichment analysis using protein-coding genes which were highly co-expressed (absolute Pearson's correlation coefficient >0.8) with it in the same module. Elliptic nodes denote the predicted functions (GO terms) for lincRNAs in the three modules.

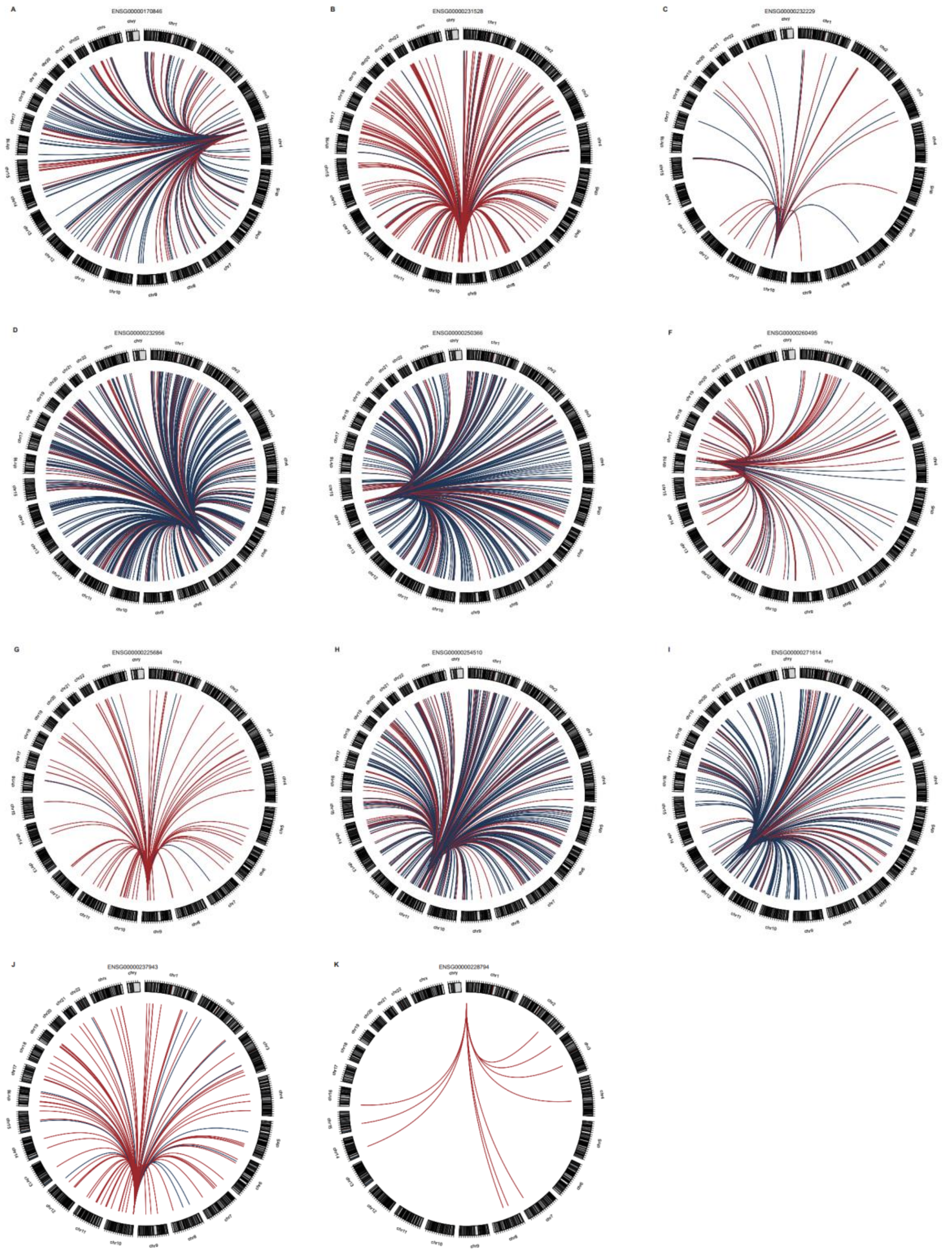

Supplementary Figure S9. Circos plot showing chromosomal positions of genes highly co-expressed (absolute Pearson's correlation coefficient >0.8) with dysregulated lincRNAs in the three dysfunctional lincRNA modules. Red links denoted correlation and blue links denoted anticorrelation.

Supplementray Tables 1-6 see online.
